# Melatonin alleviates low-sulfur stress by promoting sulfur homeostasis in tomato plants

**DOI:** 10.1038/s41598-018-28561-0

**Published:** 2018-07-05

**Authors:** Md. Kamrul Hasan, Chen-Xu Liu, Yan-Ting Pan, Golam Jalal Ahammed, Zhen-Yu Qi, Jie Zhou

**Affiliations:** 1 Department of Horticulture/Zhejiang Provincial Key Laboratory of Horticultural Plant Integrative Biology, Zhejiang University, Yuhangtang Road 866, Hangzhou, 310058 PR China; 2 Department of Agricultural Chemistry, Sylhet Agricultural University, Sylhet, 3100 Bangladesh; 3 College of Forestry, Henan University of Science and Technology, Luoyang, 471023 PR China; 4 Agricultural Experiment Station, Zhejiang University, Yuhangtang Road 866, Hangzhou, 310058 PR China; 5 Key Laboratory of Horticultural Plants Growth, Development and Quality Improvement, Agricultural Ministry of China, Yuhangtang Road 866, Hangzhou, 310058 PR China

## Abstract

Despite involvement of melatonin (MT) in plant growth and stress tolerance, its role in sulfur (S) acquisition and assimilation remains unclear. Here we report that low-S conditions cause serious growth inhibition by reducing chlorophyll content, photosynthesis and biomass accumulation. S deficiency evoked oxidative stress leading to the cell structural alterations and DNA damage. In contrast, MT supplementation to the S-deprived plants resulted in a significant diminution in reactive oxygen species (ROS) accumulation, thereby mitigating S deficiency-induced damages to cellular macromolecules and ultrastructures. Moreover, MT promoted S uptake and assimilation by regulating the expression of genes encoding enzymes involved in S transport and metabolism. MT also protected cells from ROS-induced damage by regulating 2-cysteine peroxiredoxin and biosynthesis of S-compounds. These results provide strong evidence that MT can enhance plant tolerance to low-S-induced stress by improving S uptake, metabolism and redox homeostasis, and thus advocating beneficial effects of MT on increasing the sulfur utilization efficiency.

## Introduction

Over last several decades, the practices of intensive cropping and irrigated agriculture have resulted in a drastic decrease in soil fertility across the world^1,2^. Empirical data show that approximately 40% of the world’s agricultural land is seriously degraded^3^, and the frequency of sulfur (S) deficiency ranks a close third after nitrogen (N) and phosphorus (P)^4,5^. S is an essential macronutrient required for normal metabolic processes in plants. S acts as an active structural component of amino acids, proteins and lipids, and is also vital for the biosynthesis of chlorophyll and the activation of critical enzymes and vitamins in plants. As plants serve as a chief source of organic sulfur to human diets, the increasing trend of S depletion not only reduces crop yield but also threatens human nutrition. Thus, S management towards proper uptake and homeostasis of S at different plant developmental stages and environmental conditions remains a key challenge to plant scientists^6,7^.

In plants, mainly two genes, *SULFATE TRANSPORTER1;1* (*SUT1;1*) and *SUT1;2*, are involved in S uptake from the rhizospheric soil solution^7,8^. Upon uptake, inorganic sulfate enters into the metabolic pathways by triggering ATP sulfurylase (ATPS), and is reduced to sulfide through a complex network by the activation of 5′-adenylsulfate (APS), adenylylsulfate reductase (APSR) and sulfite reductase (SiR). Finally, S is assimilated to form sulfolipids or cysteine through the catalysis of O-acetylserine(thiol)lyase (OAS-TL) or cysteine synthase and incorporated into other compounds^7,9^. Notably, the incorporation of S into thiol-containing compounds is crucial for plant growth, development and stress tolerance. Hence, the instant effects are noticed in metabolic profiles following exposure of plants to S-deprived conditions. S deficiency retards sulfate assimilation in plants, leading to significant increases in serine but reductions in glutathione, cysteine, O-acetylserine, tryptophan, chlorophyll, RNA, total protein and lipids contents^10,11^. In addition, S deficiency increases photorespiration and causes nitrogen imbalance in plants. When plants cannot acquire ample sulfate, a range of metabolic processes are consequently affected, leading to the ultimate growth inhibition^11^. Evidence from recent reports showed that phytohormones such as jasmonate, auxin, ethylene, and abscisic acid (ABA) as well as signaling molecule nitric oxide (NO) play important roles in plant responses to S deficiency^7,12^. This implies that other plant growth regulators and/or signaling molecules are also potentially involved in nutrient signaling.

Melatonin (MT; *N*-acetyl-5-methoxy tryptamine) is a ubiquitous biomolecule that primarily functions as a broad spectrum antioxidant in both animals and plants^13^. Studies on phytomelatonin suggest that MT can protect plants from oxidative stress-induced by high temperature, cold, salinity, drought, flooding, ultraviolet radiation, heavy metals and chemical pollutants^14,15^. In the past few years, there has been tremendous progress in unraveling the role of MT in plants, and the numbers of publication have undergone an exponential boost in the line of plant stress physiology^16^. Previously we showed that MT alleviates cadmium stress by stimulating thiols (cysteine, glutathione and phytochelatins) biosynthesis^17,18^, indicating a potential role of MT in S acquisition. Despite significant advances in S research in plants, the involvement of MT in the regulation of S uptake and assimilation, especially under S-deprivation remains largely unknown. Therefore, the present study was aimed to examine the effects of exogenous MT on various biochemical and physiological parameters of tomato plants under low S stress. The results showed that apart from the well-known reactive oxygen species (ROS) scavenging properties, MT could stimulate S uptake and utilization efficiency, presumably leading to an enhancement in the biosynthesis of S-compounds that confer tolerance to low-sulfur stress in tomato. Thus, MT application can be an important determinant to enhancing crop yield and quality especially in areas where S is a limiting factor for crop production.

## Results

### Melatonin enhances plant growth under low sulfur regimes

**Figure 1:**
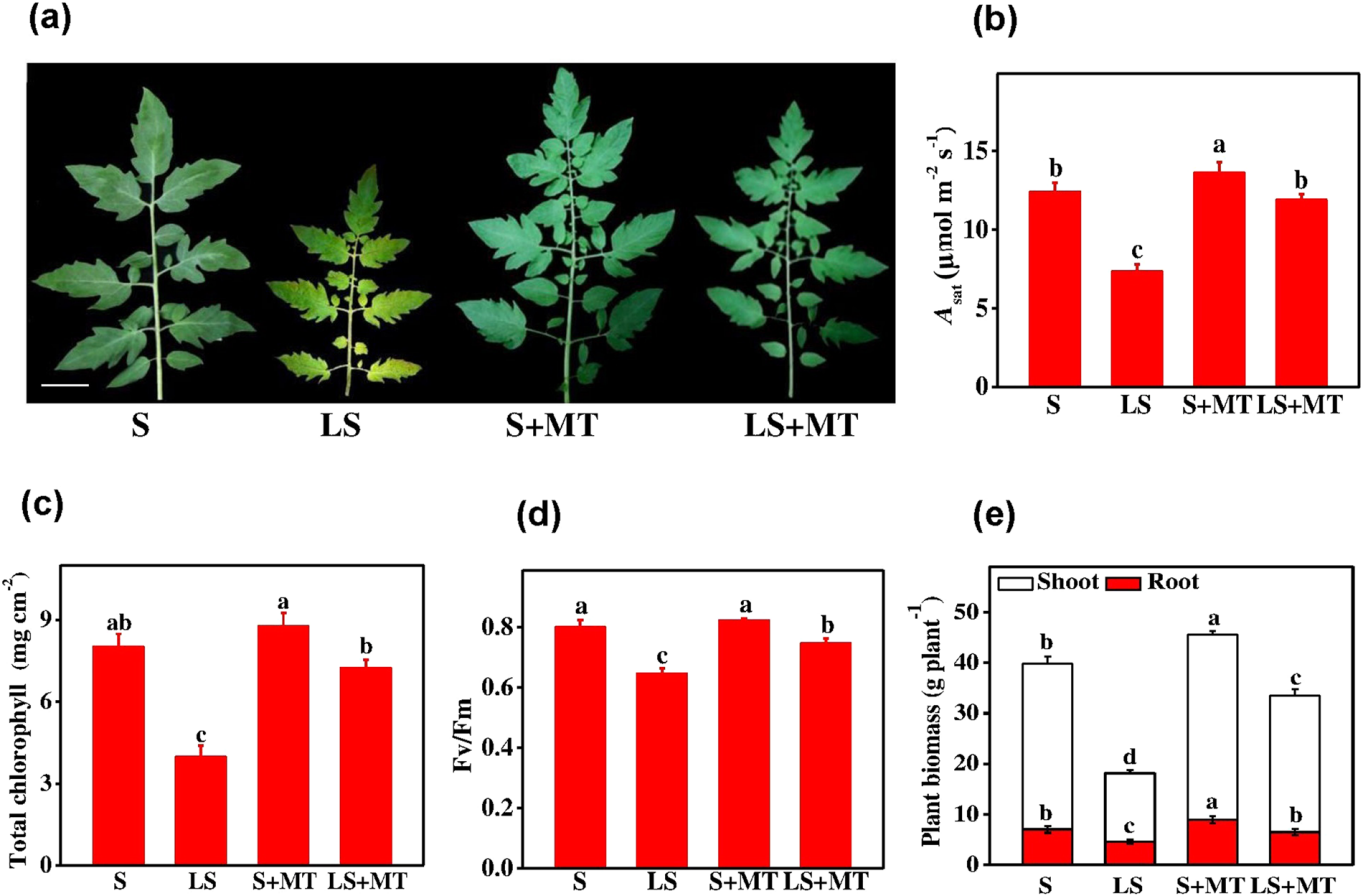
Melatonin (MT) promotes plant growth under low-S stress. (**a**) leaf phenotype, (**b**) light-saturated rate of CO_2_ assimilation (*A*sat), (**c**) total chlorophyll content, (**d**) maximum photochemical efficiency of photosystem II (*F*v/*F*m) and (**e**) biomass accumulation. Fourteen-day-old tomato plants were supplied with an optimal amount of sulfur (S) or 1/10 of optimal sulfur (LS) for 15 days; 100 µM of melatonin (MT) was applied on foliage every five days for a total of three applications. The means denoted by the same letter did not significantly differ at *P* < 0.05, according to Tukey’s test.

To investigate the role of MT in sulfur metabolism, we first administered a sulfur deficient condition by providing 1/10 sulfur into the hydroponic media as compared with the control. The results showed that sulfur deficiency caused severe growth inhibitions as evidenced by small leaf size and pale green to yellow leaf color under low sulfur treatment (Fig. 1a). Moreover, the plants grown under S-deficient conditions were spindly and short (data not shown). As compared with control, chlorophyll content, *A*sat, *F*v*/F*m and biomass accumulation decreased by 50.47%, 41.47%, 22.91% and 54.39%, respectively when plants were grown under S-deficient conditions (Fig. 1b–e). However, exogenous MT mitigated the low sulfur-induced diminutions in plant growth, chlorophyll content, *A*sat, *F*v*/F*m and biomass accumulation in tomato plants.

### Melatonin protects chloroplast and cellular macromolecules from sulfur deficiency-induced oxidative damage

**Figure 2:**
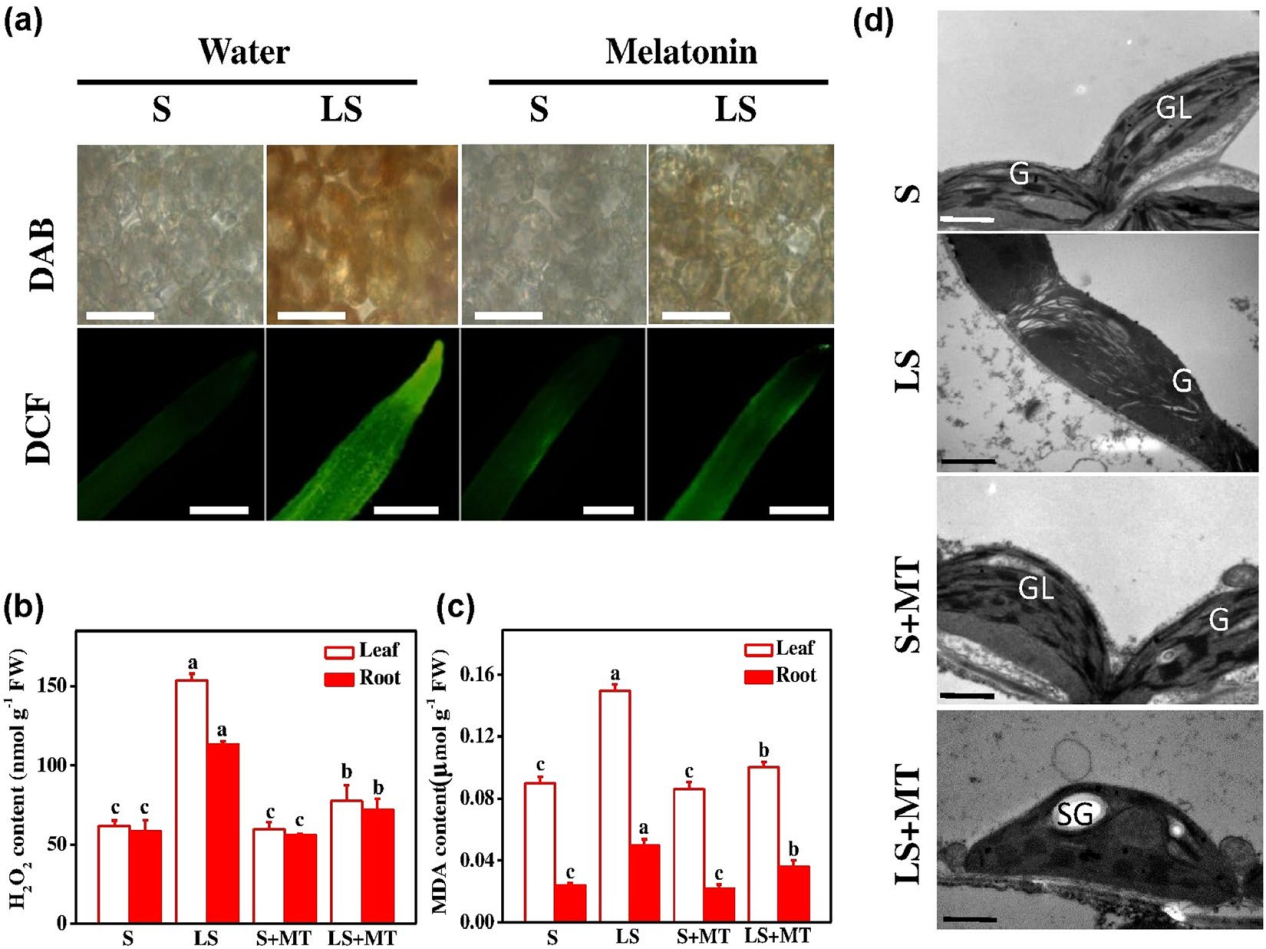
Melatonin (MT) attenuates low sulfur-induced ROS accumulation in tomato. (**a**) Histochemical staining of tomato leaves and roots with 3, 3′-diaminobenzidine (DAB) and 2′,7′-dichlorodihydrofluorescein diacetate (DCF), respectively; bar = 200 µm (upper panel) and bar = 50 µm (lower panel); (**b**) H_2_O_2_ content, (**c**) MDA content and (**d**) chloroplast ultrastructure by transmission electron microscopy (TEM) after 15 days of growth at sulfur deficient condition, bars = 0.5 µm. Fourteen-day-old tomato plants were supplied with an optimal amount of sulfur (S) or 1/10 of optimal sulfur (LS) for 15 days, and 100 µM of MT was sprayed every five days for a total of three applications. Data are presented as the means of 4 replicates. The means denoted by the same letter did not significantly differ at *P* < 0.05 according to a Tukey’s test.

To explicate the utility of exogenous melatonin in the potential amelioration of sulfur deprivation-induced oxidative stress, we focused on ROS accumulation, membrane lipid peroxidation and cell structural alterations in tomato plants (Fig. 2). Results from histochemical study with DAB and DCF staining, and biochemical quantification of H_2_O_2_ and MDA clearly showed that sulfur deprivation significantly increased ROS accumulation in leaves and roots (Fig. 2a–c). The content of H_2_O_2_ increased by 149.2% and 95.2% in leaves and roots, respectively, when plants were grown under limited sulfur conditions compared to that of control. Similarly, the MDA content, a useful indicator of ROS-induced lipid peroxidation, increased by 66.67% and 97.80% in leaves and roots in low sulfur-fed plants, respectively (Fig. 2b,c). In contrast, MT application minimized the accumulation ROS and ROS-induced lipid peroxidation in leaves and roots of tomato plants. In accordance with the low sulfur-triggered oxidative burst, accumulation of ROS caused severe cell structural alterations as shown in the TEM images (Fig. 2d). Results displayed that, deficiency of sulfur reduced not only the number and size of chloroplasts but also the volume of thylakoid membranes and grana stacking per chloroplast with irregular shape. However, melatonin supplementation markedly attenuated the low sulfur-induced structural damages to the chloroplasts. Chloroplasts in MT-treated plants were found relatively normal in shape with well-structured thylakoid membranes, parallel pattern of lamellae and increased number of grana stacking per chloroplast.

**Figure 3:**
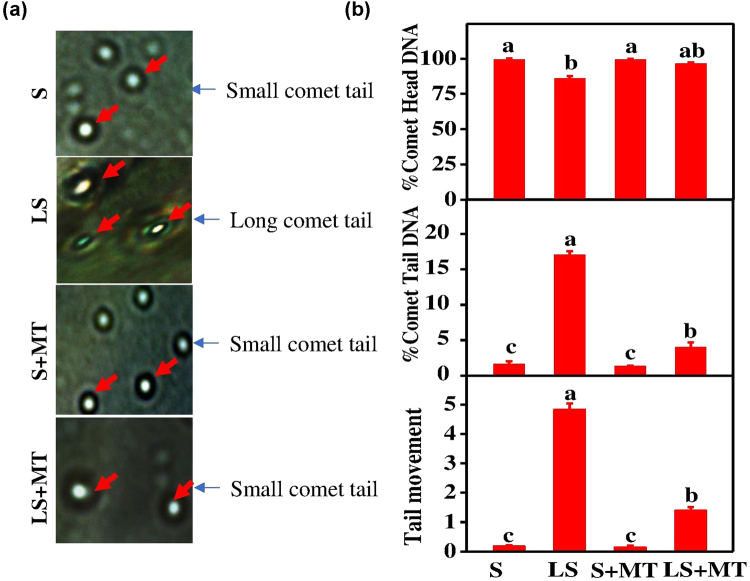
Exogenous melatonin (MT) minimizes sulfur deprivation-induced DNA damage. (**a**) Effects of melatonin with or without low S-stress on comet tail length, arrows indicate the comet tail and (**b**) percentage comet head and comet tail DNA and tail movement of DNA of tomato cells after 15-days of treatment. Fourteen-day-old tomato plants were supplied with an optimal amount of sulfur (S) or 1/10 of optimal sulfur (LS) for 15 days, and 100 µM of MT was sprayed every five days for a total of three applications. Data are presented as the means of 4 replicates. The means denoted by the same letter did not significantly differ at *P* < 0.05 according to a Tukey’s test.

To further explore the low sulfur-induced damages to cellular macromolecules, we performed comet assays that can detect DNA damage in individual plant cell (Fig. 3). We found that S deficiency resulted in the degradation of DNA in the head of the comet and thus a concomitant gain in the tail DNA, which ultimately increased the tail movement of the comet **(**Fig. 3a**)**. More precisely, S deficiency induced a 13.50% reduction in comet head DNA, which was coupled with a 10.30-fold increase in tail DNA and 23.20-fold elongation in the comet tail length compared with that of control and/or mock plants (Fig. 3b). Interestingly, exogenous MT application sharply attenuated the low S-induced DNA damage, indicating the specific role of MT in protecting cellular macromolecules from excessive ROS-induced by S deficiency.

### Melatonin promotes plant growth by improving sulfur absorption under deficient sulfur regimes

**Figure 4:**
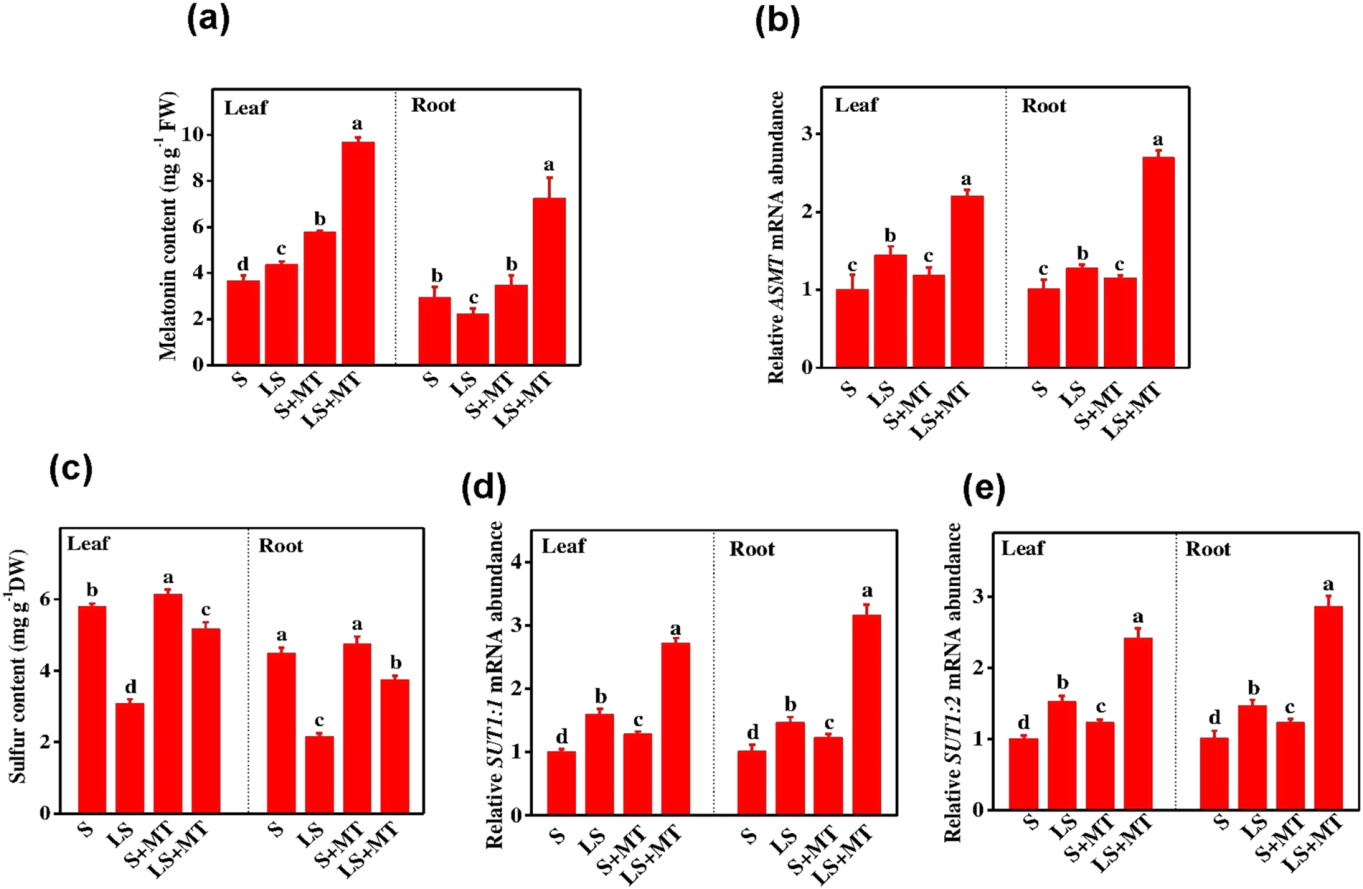
Melatonin (MT) enhances sulfur uptake and assimilation under limited S supply. (**a**) Endogenous melatonin content (**b**) relative expression of melatonin biosynthetic gene, (**c**) S content and (**d**,**e**) transcript abundance of sulfate transporter genes under sulfur deficiency in tomato. Fourteen-day-old tomato plants were supplied with an optimal amount of sulfur (S) or 1/10 of optimal sulfur (LS) for 15 days, and 100 µM of MT was supplied every five days for a total of three applications. Data are presented as the means of 4 replicates. The means denoted by the same letter did not significantly differ at *P* < 0.05 according to a Tukey’s test.

To understand whether exogenous MT-induced growth promotion was associated with the changes in endogenous MT and sulfur levels, firstly we quantified endogenous MT and sulfur contents in tomato plants (Fig. 4). MT level in tomato plants under limited sulfur conditions showed prodigious changes, as for example, MT content in leaves increased by 19.13%, whereas that in roots decreased by 25.5% compared to the plants grown in optimal sulfur conditions (Fig. 4a). However, expression of MT biosynthetic gene *ASMT* upregulated in leaves and roots (Fig. 4b). Strikingly, supplementations of MT to the S-deprived plants resulted in a dramatic increase in endogenous MT levels in both leaves and roots. Unlike endogenous MT content, sulfur deprivation decreased sulfur content by 48.32% and 52.25% in leaves and roots, respectively compared with that of normal S treatment. Foliar application of MT on sulfur-deprived plants significantly increased sulfur content by 66.13% and 74.29% in leaves and roots, respectively compared with low sulfur alone treatment (Fig. 4c). In addition, MT application onto plants grown in normal S supply also increased S content in leaves. We also monitored the expression levels of selected sulfate transporter genes, *SUT1:1* and *SUT1:2* (Fig. 4d,e), and found that the transcript levels of *SUT1:1* and *SUT1:2* in MT-treated but sulfur-deprived plants increased by 1.70- and 1.50-fold in leaves and 1.90- and /1.80-fold in roots, respectively compared with that in low sulfur only treatment, suggesting that MT plays an important role in sulfur transport under limited S supply in tomato plants.

**Figure 5:**
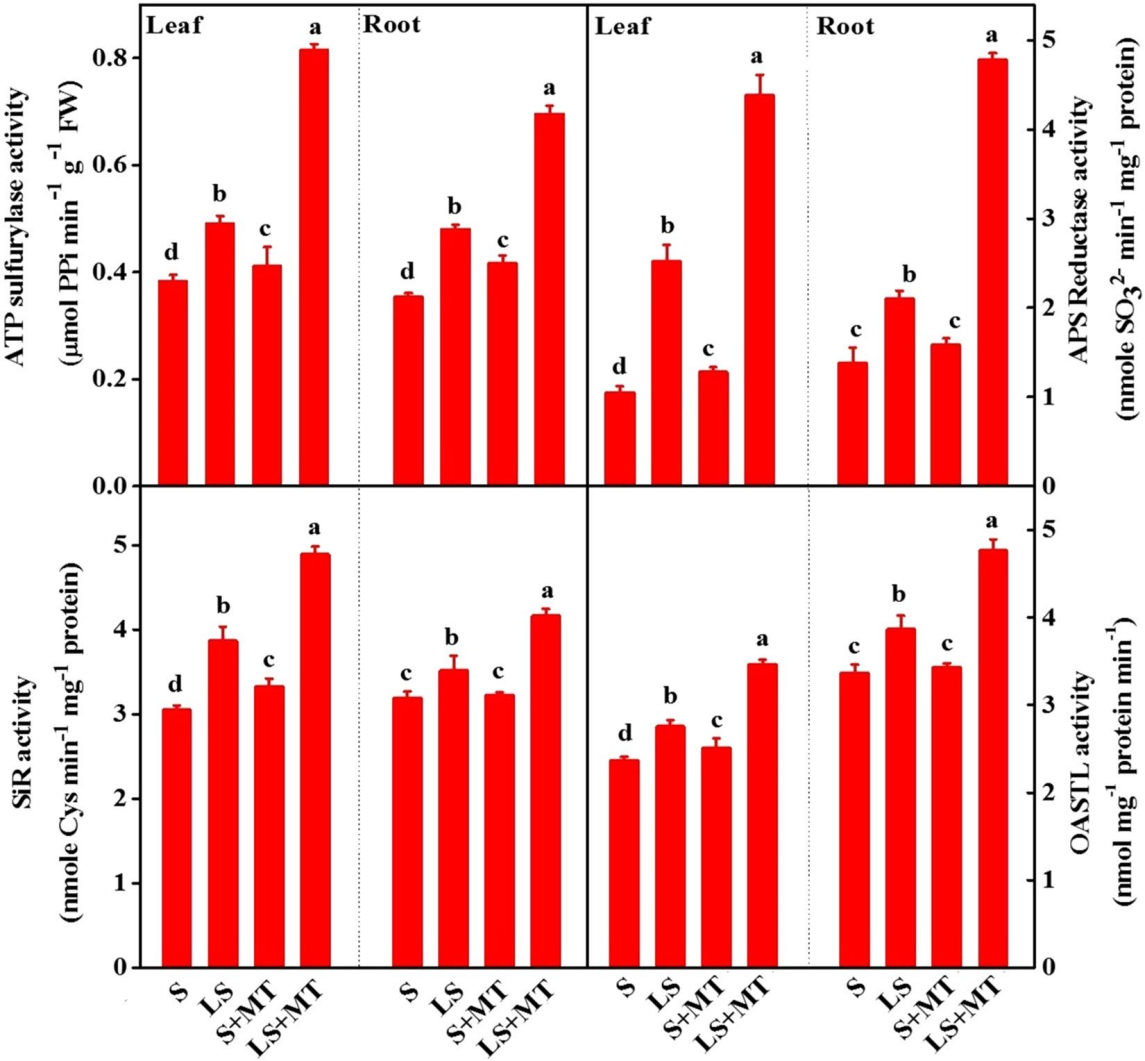
Melatonin (MT) promotes sulfur assimilation under deficient sulfur condition in tomato. ATPS, ATP sulphurylase; adenosine 5′-phosphosulfate (APS) reductase; SiR, sulphite reductase; OASTL, O-acetylserine (thiol)lyase. Fourteen-day-old tomato plants were supplied with an optimal amount of sulfur (S) or 1/10 optimal sulfur (LS) amount for 15 days, and 100 µM of MT was supplied every five days for a total of three applications. Data are presented as the means of 4 replicates. The means denoted by the same letter did not significantly differ at *P* < 0.05 according to a Tukey’s test.

**Figure 6:**
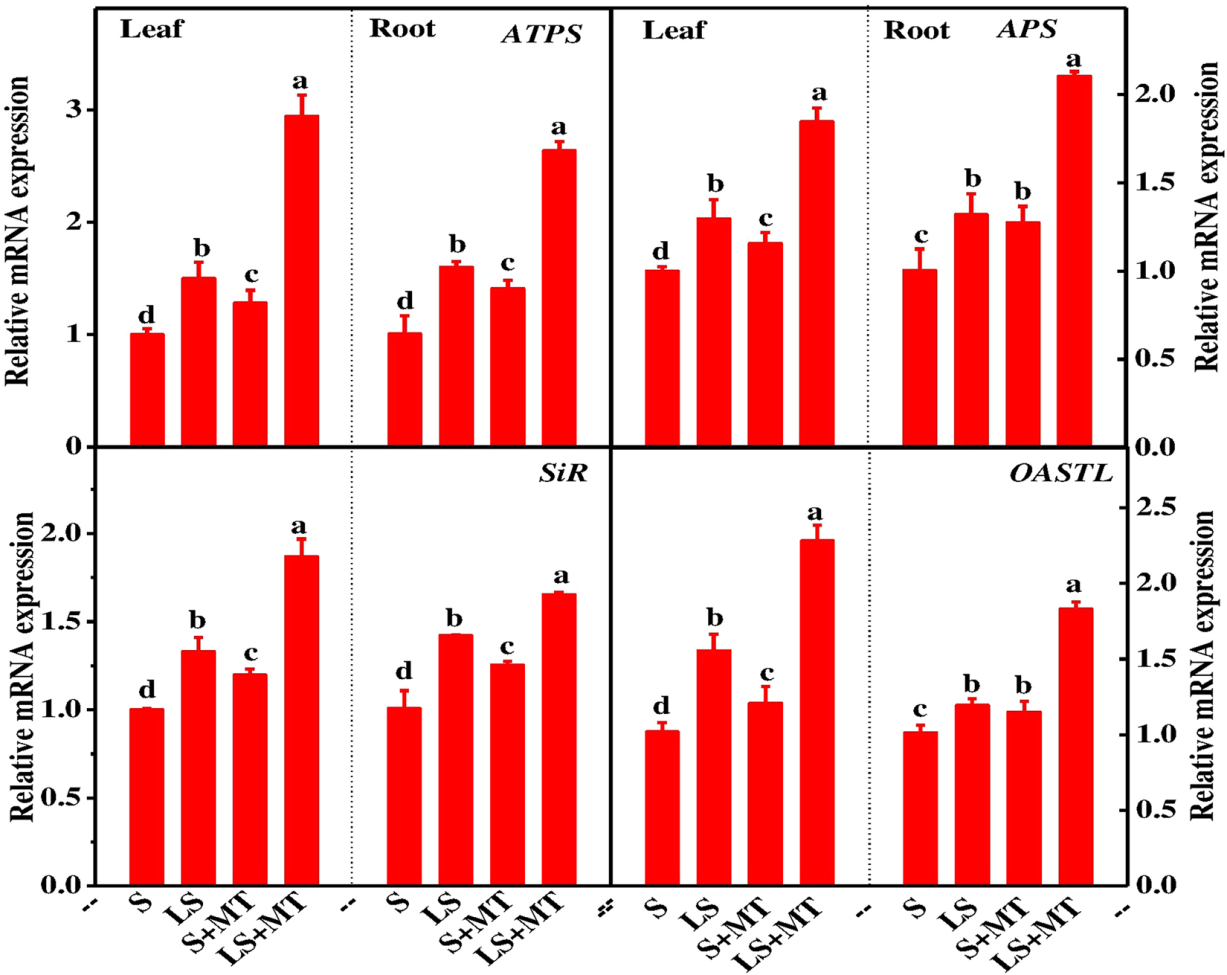
Melatonin (MT) stimulates expressions of the genes encoding step-limiting enzymes of the sulfur metabolism pathway in tomato leaves and roots. Fourteen-day-old tomato plants were supplied with an optimal amount of sulfur (S) or 1/10 of optimal sulfur (LS) for 15 days, and 100 µM of MT was supplied every five days for a total of three applications. Data are presented as the means of 4 biological replicates. The means denoted by the same letter did not significantly differ at *P* < 0.05 according to a Tukey’s test.

Upon entry into the cells, sulfur can be either stored or assimilated through the metabolic stream. To examine whether MT affects plant growth recovery by modulating sulfur assimilation, we paid attention to the activity of steps limiting sulfur metabolic enzymes (Fig. 5). Results showed that activities of all the enzymes involved in sulfur metabolic pathway increased in plants grown under sulfur-deprived media, while MT administration triggered further induction of their activities. As for example, MT supplementation increased the activities of ATPs, APS reductase, SiR and O-acetylserine(thiol)lyase (OASTL) in both leaves and roots. More precisely, MT increased those enzymes activities by 67.34%, 74.61%, 26.36% and 26.45% in leaves and 45.16%, 127.62%, 20.51% and 23.34% in roots, respectively in sulfur-deprived conditions compared with that in low sulfur alone treatment. In harmony with induced enzymatic activity, MT upregulated the transcript levels of genes encoding ATPs, APSR, SiR and OASTL enzymes in leaves and roots of sulfur-deprived plants as compared to the plants grown without MT supplementation (Fig. 6). Moreover, data of present experiment revealed that MT also increased the enzymatic activity and transcript level of related genes both in leaves and roots of plants grown in normal S supply. All these results suggest that stimulation in endogenous MT level by exogenously applied MT improved plant growth under low sulfur regimes which was possibly associated with the enhanced sulfur absorption and assimilation through selective modulation of enzymes and their genes involved in this pathway.

### Melatonin promotes biosynthesis of sulfur containing thiol compounds and 2-CP activity under deficient sulfur regimes

**Figure 7:**
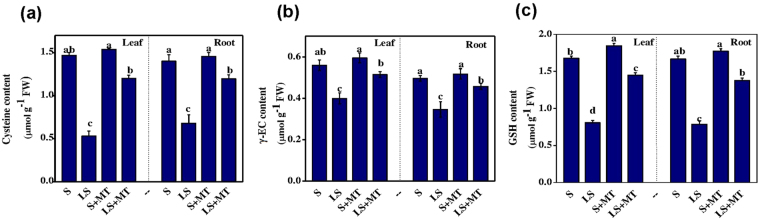
Melatonin (MT) increased the amount of sulfur-containing thiol compounds in tomato plants under low-sulfur conditions. (**a**) Cysteine, (**b**) γ-EC and (**c**) GSH contents in leaves and roots. Fourteen-day-old tomato plants were supplied with an optimal amount of sulfur (S) or 1/10 of optimal sulfur (LS) for 15 days, and 100 µM of MT was supplied every five days for a total of three applications. Data are presented as the means of 4 replicates. The means denoted by the same letter did not significantly differ at *P* < 0.05 according to a Tukey’s test.

**Figure 8:**
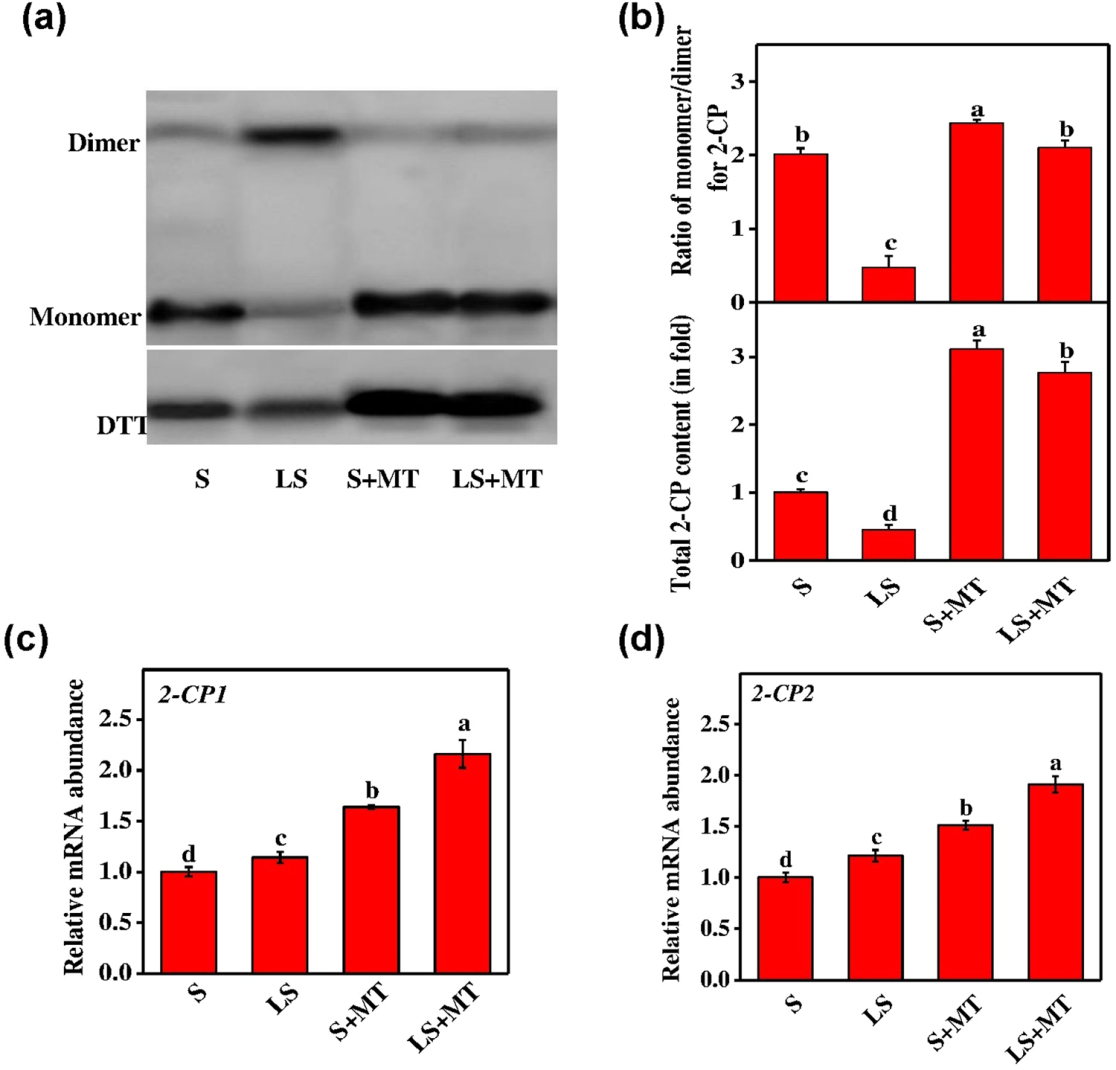
Melatonin (MT) upholds the redox state of 2-Cys peroxiredoxin (2-CP) protein in tomato leaves under low-sulfur conditions. (**a**) Total 2-CP content, (**b**) the monomer-to-dimer ratio, and (**c**,**d**) the relative transcript abundance of genes encoding the 2-CP enzyme (full-length blots are presented in Supplementary Fig. S1). Fourteen-day-old tomato plants were supplied with an optimal amount of sulfur (S) or 1/10 of optimal sulfur (LS) for 15 days, and 100 µM of MT was supplied every five days for a total of three applications. Data are presented as the means of 4 biological replicates. The means denoted by the same letter did not significantly differ at *P* < 0.05 according to a Tukey’s test.

To investigate the involvement of MT in the biosynthesis of sulfur metabolites, we quantified cysteine and its products, γ-EC and GSH, as well as 2-cysteine peroxiredoxin (2-CP) activity in tomato (Fig. 7 and y Fig. 8). Cysteine is the terminal metabolite of the reductive pathway, which represents the starting point for production of a broad variety of sulfur metabolites in plants^9^. S deprivation significantly decreased the biosynthesis of cysteine, whereas MT supplementation almost reversed that inhibition. As for example, cysteine content in sulfur-deprived plants decreased by 36.70% and 50.9% in leaves and roots, respectively. However, the administration of MT showed only 8.40% and 7.80% decrease in leaves and roots, respectively compared to the plant grown in normal sulfur supply (Fig. 7). Similarly, exogenous MT also increased contents of γ-EC and GSH in sulfur-deprived plants.

Furthermore, MT application helped plants to maintain proper redox status by stimulating 2-CP, an abundant cellular antioxidant protein as investigated by non-reducing SDS–PAGE (Fig. 8). Western blot analysis showed that the deficiency of sulfur decreased not only the total 2-CP protein but also the ratio of reduced and oxidized forms of 2-CP (monomer/dimer), reflecting the cellular redox status in chloroplast (Fig. 8a,b; full-length blots are presented in Supplementary Fig. S1). However, application of MT to the sulfur-deprived plants significantly increased total 2-CP protein and the ratio of monomer and dimer. The results indicated that MT might maintain the upstream activity of 2-CP by increased metabolic flow of cysteine as it is the main component of 2-CP. In addition, MT also upregulated the transcript levels of *CP1* and *CP2* genes encoding 2-CP protein by 2.01 and 1.60-fold, respectively in leaves compared to the plants grown under limited sulfur conditions with no MT (Fig. 8c,d). These results suggest that MT plays a critical role in cellular redox homeostasis and provides protections to cell ultrastructures under low S stress by regulating 2-CP activity through increased metabolic flow of cysteine.

## Discussion

Due to intensive cropping, soil sulfur (S) deficiency has emerged as a serious problem in sustainable crop production that not only limits crop yields but also reduces the nutritional value of agricultural produce^5,7^. In recent years, a good number of studies have implicated melatonin (MT) in boosting plant growth and stress tolerance^13^; however, the relevance of MT in S acquisition and assimilation, especially under low S regimes, remains elusive. In the present study, we revealed a strong response of tomato plants to exogenous MT under limited S regimes. We showed that exogenous MT triggered endogenous MT levels in leaves, leading to a significant improvement in plant growth, which was closely associated with MT-induced increments in S uptake, metabolism, ROS scavenging and redox homeostasis under low-S conditions in tomato plants (Figs 1–6).

Sulfur has occupied an important place after N, P and K. It is fairly essential for normal plant growth, development and vigor. Hence, S-deficiency causes severe growth retardation, leading to spindly and small plant phenotype^5^. In line with previous report, S deprivation decreased plant growth, chlorophyll content, photosystem II photochemistry (*F*v/*F*m), photosynthesis and biomass accumulation (Fig. 1), whereas exogenous MT showed a positive stress ameliorative effect on low S stress in tomato plants (Fig. 1). Previous studies also showed that low S stress decreased S-adenosyl-methionine (SAM) production leading to a reduction in chlorophyll content and an enhancement in photorespiration^19^. Moreover, S deficiency affects ribulose-1,5-bis-phosphate carboxylase/oxygenase (RuBisCO) activity and abundance of proteins involved in CO_2_ assimilation, which ultimately decrease photosynthesis and biomass accumulation in plants^10,20^ (Fig. 1). Since MT ameliorated low-S stress in tomato plants, MT-induced growth promotion could be attributed to enhanced metabolic flow of SAM.

The metabolic imbalance of S containing compounds generally leads to oxidative burst in plant cells^10^. The excessive accumulation of ROS due to S deficiency causes lipidperoxidation, damage to nucleic acids or proteins, and even cell death^21^ (Figs 2 and 3)^22^. In contrast to severe ultrastructural alterations as observed under low S stress, MT supplementation increased starch accumulation (Fig. 2c), which was consistent with the enhancement in photosynthesis following MT treatment as reported by Turk *et al*. and Sarropoulou *et al*.^23,24^. Moreover, an increased number of normal-shaped chloroplasts was observed following MT treatment under limited S supply, which may contribute to increased photosynthesis and energy production required for combating a stress^21,25^. The phenomenon of nutrient deficiency-induced changes in genetic material is very complex, and the mechanisms behind this process are not well-understood yet^21,26^. It is thought that nutrient deficiency stimulates metabolic imbalance, which ultimately induces ROS accumulation and causes DNA damage^10,27,28^. In contrast, sulfur compounds possess ability to scavenge ROS and protect organisms from DNA damage^21^. DNA damage results in various physiological effects, such as reduced protein synthesis, cell membrane destruction and damage to photosynthetic proteins, which eventually affect growth and development of the whole organism^29^. Comparative analysis of comet assay data revealed that S deficiency caused severe DNA damage in tomato plants (Fig. 3), however, exogenous MT almost reversed the S deficiency-induced DNA damage, which was in accordance with decreased ROS accumulation (Figs 2 and 3). Since MT can easily cross cellular boundaries, it is highly plausible that MT protects biological systems through direct quenching of ROS or by increased metabolic flow of sulfo-compunds, such as GSH (Fig. 7)^21,30^.

Plants take up S from soil predominantly as SO_4_^2−^ through high affinity transporters, *SULT1:1, SULT1:2*^7^. While limited S regimes increased transcript levels of the S transporter genes (*SULT1:1* and *SULT1:2*), S deficiency decreased S content, suggesting that low S-induced up-regulation in *SULT1:1* and *SULT1:2* was insufficient to minimize S deficiency in tomato seedlings (Fig. 4)^17^. Since MT content decreased along with S content in roots, a potential role of endogenous MT in S uptake and transport can easily be assumed under low S conditions. In addition, augmentation of MT significantly improved endogenous MT level and promoted S content (Fig. 4), further revealing a regulatory role for MT in S (ion) transport, which is similar as of hormonal supplementations^31,32^. Nevertheless, a role for MT in S homeostasis via regulation of miRNA cannot be ignored^33^, although such mechanisms remain poorly understood.

S assimilation is on the forefront of system biology, which provides a general platform for the biosynthesis of sulfo-compounds. In the current study, a decreased SO_4_^2−^ level in growth medium resulted in a quick drop down of internal S that differently regulated the expression of genes and enzymes involved in the assimilation pathway (Figs 5 and 6). Moreover, the deduction in SO_4_^2^ uptake led to a reduced assimilation activity as evidenced by a significant reduction in S compounds, such as cysteine, γ-glutamyl-cysteine and glutathione^11^ (Fig. 7). Notably, SO_4_^2−^ reduction takes place in plastids where it is assimilated in cysteine and incorporated to other compounds. Cysteine has SH- groups that are essential for the protein function through the formation and disruption of sulfur bridges. From our experiments, it is readily apparent that application of MT to the sulfur-deprived plants significantly increased contents of sulfo-compounds, total 2-cysteine peroxiredoxin (2-CP) protein and the ratio of reduced and oxidized forms of 2-CP (Fig. 8). 2-CP is a ubiquitous enzyme that contains two cysteine residues in both the N and C-termini^34^, and it detoxifies a broad range of peroxides through an intermolecular thiol-disulfide transition in the oxygenic environment of the chloroplast^35^. Therefore, MT-induced increases in total 2-CP and ratio of monomer to dimer reflect an improved redox status of the chloroplast (Fig. 8a,b)^35^. Additionally, recent studies of the branch-point enzymes in plant sulfur metabolism suggest that redox-regulation plays integral role in promoting S homeostasis^36^. Thus, it is logical to speculate that MT protected cells from ROS-induced damage by enhancing S assimilation and redox homeostasis as well.

Till date, how MT mediates plant response to different capricious environments remains largely unknown. Here we show that exogenous MT has a positive stimulatory effects on plant growth under low S-induced stress. MT safeguards cellular macromolecules from S deficiency-induced oxidative damage to DNA and chloroplasts. It is highly possible that MT functions as an upstream signal molecule in the sulfur metabolic pathway that controls transcriptional regulation of the genes involved in sulfur uptake and assimilation under S-deprived conditions. Moreover, MT protects cells from ROS-induced damage by regulating 2-CP and thus redox homeostasis. These results offer novel insights into the underlying mechanisms of MT-mediated regulation of S homeostasis under limited S regimes, which provide a foundation for the improvement of crop yield and quality, especially in the areas where depletion of soil S has appeared as an emerging threat to agricultural crop cultivation.

## Material and Methods

### Plant materials and experimental conditions

Seedlings of tomato (*Solanum lycopersicum* L. cv. Ailsa Craig) were grown in a combination of vermiculite and perlite (50:50, v/v), nourishing with 1/4 Hoagland’s solution. After one week culture, half of the plants were transferred to limited-S (1/10 of standard S) containing Hoagland’s hydroponics media, in which SO_4_^2−^ containing salts were replaced with an equimolar amount of Cl^−^ salts. Leaves were sprayed with 100 µM melatonin (MT) every five days for fifteen days^17^. The experiment was laid down in Completely Randomized Design (CRD) with 4 repeats, and each repeat consisted of 12 seedlings. Tomato seedlings were grown in a growth chamber under the following conditions: a mean relative humidity of 80%, a temperature of 23/20 °C (light/dark), and a photosynthetic photon flux density (PPFD) of 800 μmol m^−2^ s^−1^ with a photoperiod of 14 h light/10 h dark.

### Gas exchange, chlorophyll fluorescence and photosynthetic pigment measurements

The light-saturated rate of CO_2_ assimilation (*A*sat) was measured in the second fully expanded leaves from plant top by using IRGA (infrared gas analyzer; LI-COR 6400, Lincoln, NE, USA), a portable photosynthesis system. The assessment were executed between 8.00–11.00 am maintaining the air relative humidity, temperature, CO_2_ concentration, and PPFD, at 85%, 25 °C, 400 μmol mol^−1^, and 1000 μmol m^−2^ s^−1^, respectively. The maximum quantum efficiency of photosystem II (PSII), expressed as *F*v/*F*m, was also determined in the second leaves from plant top after 30 min of dark treatment with an imaging pulse amplitude-modulated (PAM) fluorimeter (IMAG-MAXI, HeinzWalz, Effeltrich, Germany), as discussed by Zhou *et al*.^37^. Leaf chlorophylls (Chla and Chlb) and carotenoids (Carts) were extracted in 80% acetone and measured spectrophotometrically at 470, 646, and 663 nm wavelength, respectively^38^.

### Determination of H_2_O_2_ and lipid peroxidation

The H_2_O_2_ accumulation in the leaves was histochemically detected by staining with DAB (3,3′-diaminobenzidine, Aladdin Reagent Co. Ltd., Shanghai, China) according to the method described by Christensen *et al*.^39^. Soon after plucking, the leaves were submerged in 1 mg mL^−1^ DAB solution (pH 3.8) and incubated for 6 h under light at 25 °C. The accumulation of H_2_O_2_ in the roots was detected by DCHF-DA (2′, 7′-dichlorodihydrofluorescein diacetate, Sigma-Aldrich, St. Louis., MO, USA) staining^40^. For the biochemical determination of H_2_O_2_ and MDA content, 0.3 g fresh leaves and roots samples were homogenized with ice cool extraction buffer and assessed spectrophotometrically as described previously^38^.

### Cell ultrastructure study by transmission electron microscopy (TEM)

Tomato leaves were collected after 15 days of low S treatment. Immediately after harvesting,, samples were cut with a sharp razor blade into 2-3 mm fragments and fixed in 0.1 M PBS (sodium phosphate buffer, pH7.4) solution with 2.5% glutaraldehyde (v/v) and kept at room temperature overnight. Afterward, the leave samples were washed with the same PBS solution and post-fixed in 1% OsO_4_ (osmium (VIII) oxide) for 1 h and washed again with the same buffer. The samples were then dehydrated in 50, 60, 70, 80, 90, 95 and 100% of ethanol for 15–20 min intervals and finally 20 min in absolute acetone. Next leave specimens were infiltrated and embedded overnight in Spurr’s resin. Finally, ultra-thin sections (80 nm) were prepared by heating the specimens at 70 °C for 9 h and mounted on copper grids for observation using a transmission electron microscope (JEOLTEM-1230EX) at an accelerating voltage of 60.0 kV.

### Single cell gel electrophoresis assay (comet assay)

Comet assay of the leaves of tomato plants were performed according to the method described by Sakamoto *et al*.^41^. The generated images of ethidium bromide-stained comets were taken by using BX61v microscope (Olympus Co., Tokyo, Japan) equipped with a digital CCD camera (Olympus Co., Tokyo, Japan). The captured comets were examined using CASP software (http://www.casp.of.pl/).

### Determination of S contents

For the determination of S content, plant samples were digested with di-acid mixture (HClO_4_ and HNO_3_ mixture; v:v = 1:3) at 180 °C, and following the digestion, 2 mL of diluted HNO_3_ (distilled water:concentrated HNO_3_ = 1:1) was added to the samples and then washed with distilled water at least three times. The resulting liquid was collected and diluted to a constant volume and S concentration was measured by the turbidimetric method^42^.

### Extraction and quantification of plant endogenous melatonin

MT contents were determined according to Li *et al*.^18^. In brief, 0.3 g fresh samples were crushed in liquid nitrogen and homogenized in 3 mL of methanol containing 50 ng mL^−1^ [_2_H^6^]-melatonin (M215002, Toronto Research Chemicals Ltd., Toronto, Ontario, Canada) as an internal standard. The homogenate was shaken overnight in the dark at 4 °C and then centrifuged at 15,000 g for 10 min. The supernatant fraction was removed to a new tube, and the pellet was then re-extracted and mixed with the supernatant fraction. The samples was then eluted through a Sep-Pak C^18^ cartridge (WAT020805, Waters, Milford, MA, USA) to remove the polar compounds and dried under nitrogen. Dried samples were resuspended in 0.5 mL methanol (70%), and subjected to analysis by HPLC electrospray ionization/MS-MS at 40 °C, coupled to an Agilent 6460 triple Quad LC/MS and Agilent-XDB C^18^ column (3.5-µm, 150-mm × 2.1-mm, Agilent Technologies, Frankfurt, Germany). The [_2_H^6^]-MT was also quantified as an internal standard to estimate the recovery rate^18^.

### Assay of ATP-sulfurylase activity

For the determination of ATP-sulfurylase activity in tomato plant, 0.3 g fresh samples (leaf and root) were crushed with chilled mortar and pestle using ice cool buffer containing 10 mm Na_2_EDTA, 20 mm Tris–HCl (pH 8.0), 2 mm dithiothreitol and 0.01 g ml^−1^ insoluble polyvinylpyrollidone. Afterward, the homogenate was strained through gauze and centrifuged at 20 000 g for 10 min at 4 °C. The supernatant fraction was transferred to a new tube and used for the *in vitro* ATP-sulfurylase assay as molybdate-dependent formation of pyrophosphate^43^. In the first aliquot, the reaction was started by adding 0.1 ml of the sample extract to 0.5 ml of the reaction mixture that contained 7 mm MgCl_2_, 5 mm Na_2_MoO_4_, 2 mm Na_2_-ATP and 0.032 U ml^−1^ of sulphate-free inorganic pyrophosphate in 80 mm Tris–HCl buffer (pH 8.0). In the second aliquot, the sample extract was the same but in the reaction mixture, Na_2_MoO_4_ was absent. Both the aliquots were incubated at 37 °C for 15 min and spectrophotometrically determined the phosphate, and calculated ATP-sulfurylase activity from the difference between the two figures^43^.

### Determination of adenosine 5′-phosphosulfate (APS) reductase and sulfide reductase (SiR) activities

To determine the APSR activity, protein was extracted from the tissues in 100 mM Tris-acetate buffer (pH 8.0) with 500 mM sulfate. The extracted protein samples were desalted by passage through Sephadex G-25 columns and then equilibrated with the same buffer used for the extraction. An APR activity kinetic assay employing an APS regenerating system with ATPS enzyme was performed in a reaction mixture containing 100 mM Tris-acetate buffer (pH 8.0) with 500 mM sulfate, 50 mM MgATP and 50 mM GSH. The purple color that developed from the fuchsin dye reagent, which indicated the sulfite level, was measured spectrophotometrically at the 570-nm wavelength^44^. Similarly, the kinetic assay of SiR activity was performed in desalted proteins using a reaction mixture containing 25 mM phosphate buffer (pH 7.5), 6 mM *O*-acetyl-L-serine, 6 mM sodium dithionite, 1 U of OAS-TL and methyl viologen were added prior to reaction. The reaction was stopped after 30 min with a 1/4 volume of 10% trichloroacetic acid (TCA) and finally cysteine content was determined as described by Brychkova *et al*.^45^.

### Assay of O-acetylserine (thiol)lyase (OASTL) activities

Fresh leaf and root samples (0.3 gm) were homogenized with chilled mortar and pestle using ice cool extraction buffer containing 250 mM potassium phosphate (pH 8.0), 0.5 mM EDTA, and 10 mM 2-mercaptoethanol. The activity of OASTL was determined in sample extract by using the reaction mixtures containing 50 mM potassium phosphate (pH 8.0), 5 mM Na2S and 12.5 mM OAS. After incubation at 30 °C for 10 min and the reaction was terminated by the addition of 1/5 volumes of 7.5% (w/v) trichloroacetic acid. The cysteine produced in the reaction was measured spectrophotometry at 560 nm by using the acid-ninhydrin method^46^. Protein concentrations were measured according to the previously described method using BSA as a standard^46^.

### Determination of thiol compounds by HPLC

For the determination of thiol compounds, 1 g of plant samples were ground with liquid nitrogen, and homogenized with 1 mL of extraction buffer containing 6.3 mM DTPA (diethylene triamine pentaacetic acid) and 0.1% (v/v) TFA (trifluoroacetic acid) as described by Hasan *et al*.^17^. The homogenates were centrifuged at 12,000 g for 10 min at 4 °C and the supernatants were collected. The samples were then derivatized with monobromobimane (mBBr) according to the method described by Minocha *et al*.^47^. After derivatization, samples were filtered with 0.45 µm nylon syringe filters and subjected to HPLC analyses. The thiol compounds; cysteine, γ–EC and GSH were separated by HPLC equipped with a fluorescence detector set at 380 and 470 nm wavelengths, a Phenomenex Synergi Hydro-RP C^18^ column (4 µm particle size, 100 mm × 4.6 mm) and a C^18^ SecurityGuard^TM^ (5 µm, 4 mm × 3 mm) cartridge guard column^47^.

### Western blot analysis of 2-Cys peroxiredoxin

Total protein was isolated from the leaf samples with a protein extraction buffer containing 100 mM HEPES (pH 7.5), 5 mM EDTA, 5 mM EGTA, 10 mM Na_3_VO_4_, 10 mM NaF, 50 mM β-glycerophosphate, 1 mM phenylmethylsulfonyl fluoride, 10% glycerol, and 7.5% polyvinylpolypyrrolidone supplemented with 10 mM *N*-ethylmaleimide (NEM) for thiol-blocking as described by Cheng *et al*.^35^. To find the total 2-CP, we used β-mercaptoethanol (β-ME), despite the presence of NEM, and 1% dithiothreitol (DTT) to maintain the reduced state. After the extraction, 15 μg protein samples accomplished with 5 × loading buffer [225 mM Tris-HCl (pH 6.8), 5% SDS, 50% glycerol, and 0.05% bromophenol blue] were separated by using 12% SDS-PAGE, and the redox state of the 2-CP was spotted by western blot analysis with a polyclonal antibody against 2-CP (Beijing Protein Innovation, Beijing, China). After incubation with a horseradish peroxidase-linked secondary antibody (Cell Signaling Technology, Boston, MA, USA). The generated blot complexes were visualized by using an enhanced chemiluminescence kit (Perkin Elmer, Wellesley, MA, USA). The band intensity was measured by using Quantity One software (Bio-Rad Laboratories, Hercules, CA, USA).

### Extraction of total RNA and quantitative real-time PCR (qRT-PCR) analysis

Total RNA was extracted from the tomato leaf and root tissues using Tiangen RNA extraction kit (Tiangen, Shanghai, China). Aliquots (1 µg) of resulting total RNA were reverse-transcribed to generate cDNA using a ReverTra Ace qPCR RT Kit (Toyobo, Japan), according to manufacturer’s instructions. The qRT-PCR analysis was performed using the Takara SYBR Green PCR Master Mix (Takara, Tokyo, Japan) on an AB StepOnePlus™ Real-Time PCR System (Applied Biosystems, Foster City, CA, USA) under the default thermal cycling conditions with an added melting curve. Gene-specific primers for qRT-PCR were designed based on the published sequences and the primers corresponding to the genes *ASMT, SUT1:1, SUT1:2, ATPS, APS, SiR, OASTL, CP1*, and *CP2*; the marker gene *UB*I3 and *actin* were used as internal control. Gene-specific primer pairs are presented in Supplementary Table S1. The PCR conditions were similar to those described previously^17^. The relative quantification of mRNA levels were calculated according to Livak and Schmittgen^48^.

### Statistical analysis

The experiment was laid down in Completely Randomized Design (CRD) with 4 replicates, and each replicate consisted of 12 seedlings. All data were subject to the normality test by the Shapiro-Wilk test and the means were compared for significant difference using a Tukey’s test (*P* < 0.05). Each biochemical assay had at least four replicates and data were expressed as the means ± standard deviations (SD) and statistical analyses were performed using Data Processing System (DPS) statistical software package.

## Electronic supplementary material

Supplementary Information
