# Supplementary material for: Melatonin alleviates low-sulfur stress by promoting sulfur homeostasis in tomato plants
Source: Sci Rep. 2018 Jul 5;8:10182. doi: 10.1038/s41598-018-28561-0 (PMC6033901; doi:10.1038/s41598-018-28561-0)

Melatonin alleviates low-sulfur stress by promoting sulfur homeostasis in tomato plants

Md. Kamrul Hasan[1,2], Chen-Xu Liu[1], Yan-Ting Pan[1], Golam Jalal Ahammed[3], Zhen-Yu Qi[1,4*], Jie Zhou[1,5*]

[1]Department of Horticulture/Zhejiang Provincial Key Laboratory of Horticultural Plant Integrative Biology, Zhejiang University, Yuhangtang Road 866, Hangzhou, 310058, PR China;

[2]Department of Agricultural Chemistry, Sylhet Agricultural University, Sylhet, 3100, Bangladesh;

[3]College of Forestry, Henan University of Science and Technology, Luoyang, 471023, PR China;

[4]Agricultural Experiment Station, Zhejiang University, Yuhangtang Road 866, Hangzhou, 310058, PR China;

[5]Key Laboratory of Horticultural Plants Growth, Development and Quality Improvement, Agricultural Ministry of China, Yuhangtang Road 866, Hangzhou, 310058, PR China

Supplementary information

Supplementary Table-S1 Primer sequences used for the qRT-PCR of selected genes.

Gene name	Functional annotations	Primer sequences (5′-3′)	Accession numbers
ASMT	Melatonin biosynthesis	F:TTTGTTAGTGTGCCGAAAGC; R: ACCGCTTCGTAGCAATTCTT	Solyc03g080180.2.1
SUT1	Sulfate anion transporter *SUT1;1*	F:GATATTGCGATAGGGCCAGT; R: GCAAGACGCTGATACTCGTG	Solyc10g047170.1.1
SUT2	Sulfate anion transporter *SUT1;2*	F:CCTGTTGCTGTTGTATCGCT; R: CAAATGCAGCCTGGAAAGTA	Solyc12g056920.1.1
ATPS	ATP sulfurylase (sulfate adenylyltransferase)	F: TCGTCTTGAAGATGGCTCTG; R: GGTCGAGCCACCAATTAGAT	Solyc03g005260.2.1
APS	adenylyl-sulfate reductase	F: TGAGAAGTTGGCTGGTTCTG; R: ATGGCCTTTGAAGAGTGCTT	Solyc02g080640.2.1
SiR	Sulfite reductase - ferredoxin dependent	F:AAAGAACCCTGGTGGTGAAG; R:CATGCAGCTGAAAGGTCTGT	Solyc11g065620.1.1
OASTL	O-acetylserine (thiol)lyase	F: ATGCTGAGGAGAAGGGCTTA; R: CCATGAATGCCAATCCTACA	Solyc08g014340.2.1
CP1	Alkyl hydroperoxide reductase /Thiol specific antioxidant	F: AAAGTCCGGTGGTCTAGGTG; R: GTGTAAGCCACGAGAAACCA	Solyc10g082030.1.1
CP2	Alkyl hydroperoxide reductase /Thiol specific antioxidant	F: ACCATGCATTTCTGCAAGAG; R: CCCTCAACCCTTCTCCACTA	Solyc01g007740.2.1
UBI3	Ubiquitin3	F: AGTCCACTCTCCATCTCGTG; R: CTCAGCATTAGGGCACTCCT	Solyc01g056940.2.1
Actin	marker gene	F: TGGTCGGAATGGGACAGAAG; R: CTCAGTCAGGAGAACAGGGT	Solyc03g078400.2.1

Supplementary Figure S1 (a) full length blot for the ratio of monomer-to-dimer of 2-CP protein

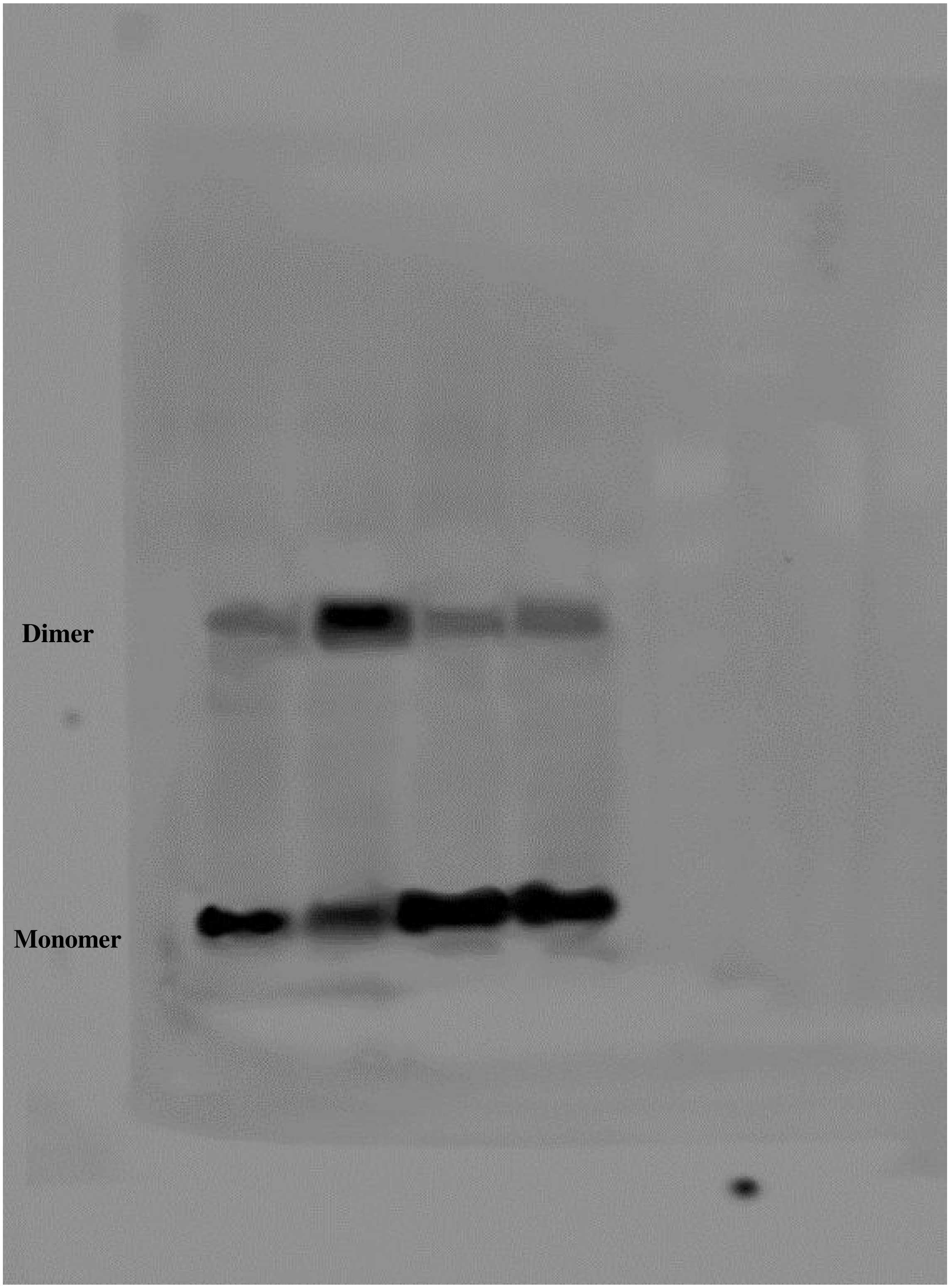

(b) Full length blot of total 2-CP protein

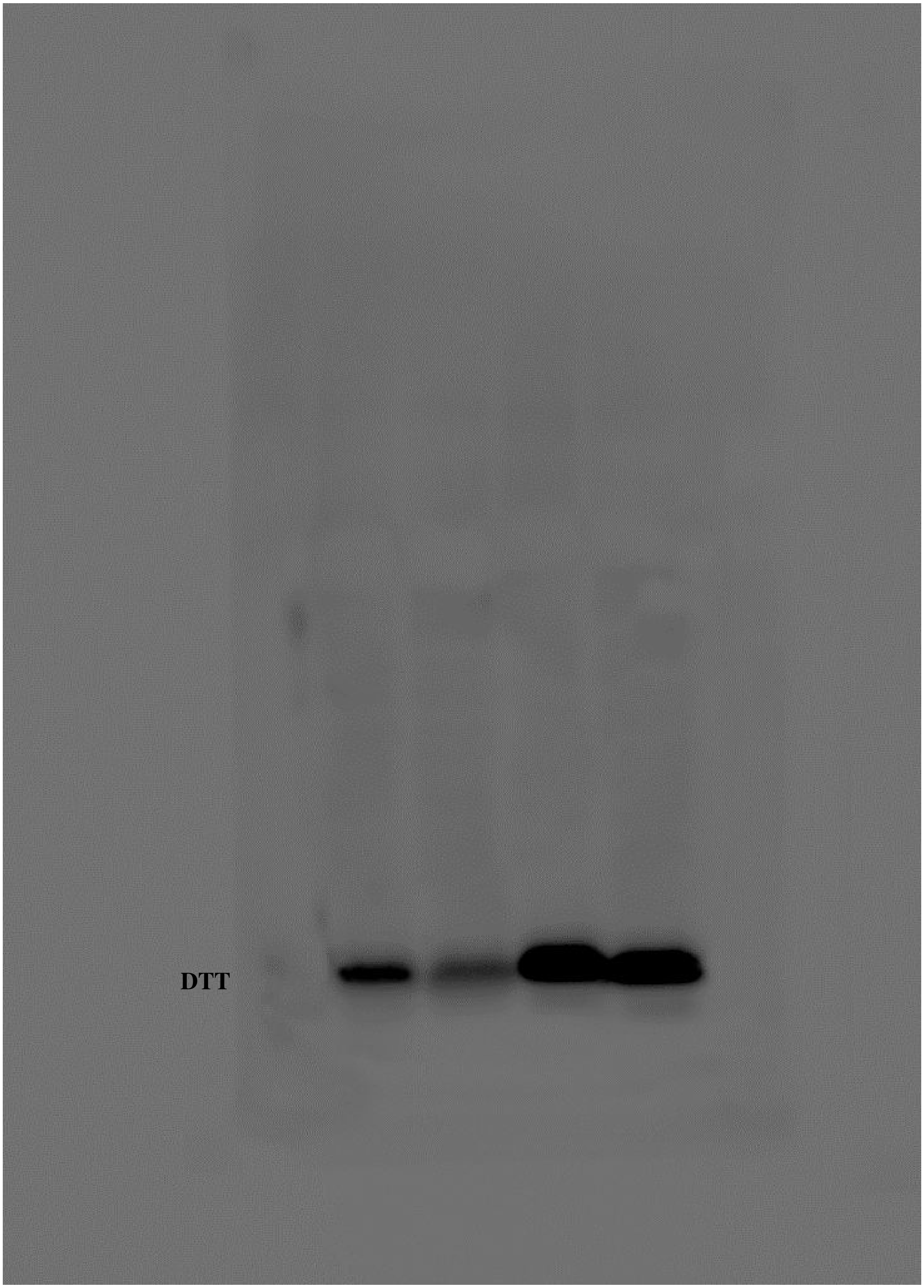

Supplement: Supplementary file 1 — Supplementary Information [file 41598_2018_28561_MOESM1_ESM.pdf]
